# Supplementary material for: Studying Factors Affecting Success of Antimicrobial Resistance Interventions through the Lens of Experience: A Thematic Analysis
Source: Antibiotics (Basel). 2022 May 10;11(5):639. doi: 10.3390/antibiotics11050639 (PMC9137464; doi:10.3390/antibiotics11050639)
Supplement: Supplementary file 1 [file antibiotics-11-00639-s001.zip › antibiotics-1694036-supplementary.pdf]

Supplementary material: Studying factors affecting success of antimicrobial resistance interventions through the lens of experience: a thematic analysis

Table S1: Standard Reporting for Qualitative research (SRQR) and reference 36 of the main manuscript [1].

Number	Topic	Manuscript	Page
**S1**	Title	x	1
**S2**	Abstract	x	1
**S3**	Problem formulation	x	2
**S4**	Purpose or research question	x	2
**S5**	Qualitative approach and research paradigm	x	13 and Table S2
**S6**	Researcher characteristics and reflexivity	x	13 and Table S2
**S7**	Context	x	2
**S8**	Sampling strategy	x	13 and Table S2
**S9**	Ethical issues pertaining to human subjects	x	14 and Table S2
**S10**	Data collection methods	x	13and Table S2
**S11**	Data collection instruments and technology	x	13 and Table S2
**S12**	Units of study	x	13 and Table S2
**S13**	Data processing	x	13 and Table S2
**S14**	Data analysis	x	13 and Table S2
**S15**	Techniques to enhance trustworthiness	x	13 and Table S2
**S16**	Synthesis and interpretation	x	2-13
**S17**	Links to empirical data	x	Tables and Figures
**S18**	Integration with prior work	x	11-13
**S19**	Limitations	x	13
**S20**	Conflict of Interest	x	14
**S21**	Funding	x	14

Supplementary material: Studying factors affecting success of antimicrobial resistance interventions through the lens of experience: a thematic analysis

Table S2: Thematic analysis iterative process following the methodology described elsewhere [2]**,** reference number 17 in the main manuscript, and adapted to our study about obstacles and success factors in AMR interventions.

1. Familiarization
Reading and reviewing answers several times, noting down details.
2. Initial Coding
Bottom-up coding: detailed and specific ideas extracted from the answers. Coding was performed using MAXQDA v.2020, a computer assisted qualitative data analysis software, without a pre-existing coding frame and, therefore, was inductive, allowing the data to drive the themes (data-driven). Re-occurrences, peculiarities grouped in different codes. Very detailed codes. A series of workshops with experts in the field was conducted as part of the AMResilience project and they gave us feedback.
3. Identify potential themes
After feedback, the aim of this phase was to inductively code and generate more general themes to the differently ways of answering. To clearly conceptualize the research question and to prevent bias from the main researcher whose experience involves clinical microbiology and epidemiology, three more co-authors independently coded responses to assess interpersonal consistency and discrepancies were discussed to clarify criteria. The same text could be related to different codes if more than one idea was underlying. In general, there was agreement between ideas extracted and discrepancies were discussed to assess slightly different points of view until final consensus. At this point, classification of themes was decided to be done in 3-levels: the highest level was defined as the theme or main topic, inside this one a second optional level of categories, and, the lowest level was defined as sub-themes. The third level or sub-theme was the most detailed to not miss any idea but could be clustered in other sub-theme afterwards. A theme was defined as the main explicit and clear tacit idea behind the participant answer and could be broken into sub-themes which were detailed specific factors related to the main theme. A category was a (optional) middle classification where sub-themes could be related in a smaller group. First themes extracted from answers.
4. Reviewing themes
Reviewing codes in phase 3. Requirements for extracting general patterns often need a certain amount of data and repetition. Otherwise, they were anecdotal and subsequently excluded. Frequency was counted as interventions mentioning a particular theme, category, or sub-theme independently of the number of times repeated on each data item. Redundancies were included to not miss information, but if present in the same data item they were counted as one. Clusterization of codes (themes and subthemes) with similar or equal connotations. Simplified thematic map.
5. Defining themes
Defining key themes and subthemes which have similar meaning or same meaning but different perspectives. Third refining of themes. Factors seen as key components for positive outcomes (either because factors were seen satisfactory or the opposite, or lack of, obstructive) were re-organized and clustered together (total frequency) although each contribution was considered (partial frequency). Integration and meaning with the conducted research question: causes for success of interventions tackling antimicrobial resistance.
6. Thematic analysis report
Outcomes of the captured themes when addressing AMR: meaning, assumptions and implications of each theme, report most mentioned themes (frequency) and sub-themes**.** Qualitative relation between sub-themes were mapped using the online software draw.io and sub-themes were connected when a respondent overlapped or implied tacitly a certain overlap sub-themes to have a successful intervention in that case. Background (sectors, economic income, funding) checked and compared to find differences between themes and sub-themes.

Supplementary material: Studying factors affecting success of antimicrobial resistance interventions through the lens of experience: a thematic analysis

Table S3: Published articles included in the thematic analysis.

Title of the publication	Year	Reference
Possible impact of the “yellow card” antimicrobial scheme on meat inspection lesions in Danish finisher pigs	2013	[3]
Control of Fluoroquinolone Resistance through Successful Regulation, Australia	2012	[4]
Matrix-assisted laser desorption ionization time-of-flight mass spectrometry and PCR-based rapid diagnosis of Staphylococcus aureus bacteraemia	2013	[5]
National laboratory-based surveillance system for antimicrobial resistance a successful tool to support the control of antimicrobial resistance in the Netherlands	2017	[6]
Implementation of treatment guidelines to support judicious use of antibiotic therapy	2010	[7]
Trends in Antibiotic Utilization in Vancouver Associated With a Community Education Program on Antibiotic Use	2010	[8]
Contribution of antimicrobial stewardship programs to reduction of antimicrobial therapy costs in community hospital with 429 Beds	2014	[9]
The influence of a sustained multifaceted approach to improve antibiotic prescribing in Slovenia during the past decade findings and implications	2015	[10]
How a smiley protects health: A pilot intervention to improve hand hygiene in hospitals by activating injunctive norms through emoticons	2018	[11]
Impact of Quinolone Restriction on Resistance Patterns of Escherichia coli Isolated from Urine by Culture in a Community Setting	2009	[12]
Danish Integrated Antimicrobial Resistance Monitoring and Research Program	2007	[13]
Prescribed medications and pharmacy interventions for acute respiratory tract infections in Swiss primary care	2009	[14]
Decreased Resistance to Broad-Spectrum Cephalosporin in Escherichia coli from Healthy Broilers at Farms in Japan After Voluntary Withdrawal of Ceftiofur	2015	[15]
“Antibiotics Are Not Automatic Anymore”—The French National Campaign To Cut Antibiotic Overuse	2009	[16]
National Antimicrobial Resistance Monitoring System Two Decades of Advancing Public Health Through Integrated Surveillance of Antimicrobial Resistance.	2017	[17]
Comparison of annual and regional variation in multidrug resistance using various classification metrics for generic Escherichia coli	2018	[18]
The challenges and successes of implementing a sustainable antimicrobial resistance surveillance programme in Nepal	2014	[19]
Impact of restriction of third generation cephalosporins on the burden of third generation cephalosporin resistant K. pneumoniae and E. coli in an ICU	2009	[20]
An investigation of antifungal stewardship programmes in England	2017	[21]
Reducing Antimicrobial Usage in Pig Production without Jeopardizing Production Parameters	2016	[22]
Scottish Antimicrobial Prescribing Group (SAPG) development and impact of the Scottish National Antimicrobial Stewardship Programme	2011	[23]

Supplementary material: Studying factors affecting success of antimicrobial resistance interventions through the lens of experience: a thematic analysis

Table S4: Themes hindering interventions or their implementation not related to mentioned success factors.

Theme	Sub-theme [prevalence]	Meaning and assumptions	Implications	Quotes
**Capacity - Resources [7/21]**	Personnel<br><br>[4/21]	Lack of personnel. Intervention may not be well or fully implemented due to personnel shortage. Available personnel may be working overtime or results of the intervention may be delayed.	Hiring more personnel to carry out the intervention which will probably need more funding for hiring employees. Or, it may need better planning or management to adequate available personnel to the intervention improve implementation (e.g. realistic workload estimations for personnel available)	"available personnel" or "Insufficient number of technicians regarding the increasing number of samples received per year (increase of 5% of the activity, but decreased budget)"
	Time overloaded<br><br>[4/21]	Intervention was carried out by staff already working full-time and with other duties. Interventions may not be carefully carry out (stress, multitasking)	Hiring more personnel to carry out the intervention or relieve duties from personnel available to carry out the intervention. Probably will need more resources (e.g. funding for employees)	"available time" or "lack of time and other commitments (e.g. other clinical work / meetings etc)"
	General or unspecific resources<br><br>[2/21]	Lack of any type of resources that hinder the implementation or application of the intervention, or it may stop it.	It will probably need more funding (e.g. buy enough resources to have reserve) or better planning / management to improve implementation (e.g. better estimations of quantity of resources needed for a period)	"Resources" or "shortage of resources in the routine diagnostic lab"

References:

1. O'Brien, B.C.; Harris, I.B.; Beckman, T.J.; Reed, D.A.; Cook, D.A. Standards for reporting qualitative research: A synthesis of recommendations. *Acad. Med.* **2014**, *89*, 1245–1251.

2. Braun, V.; Clarke, V. Using thematic analysis in psychology. *Qual. Res. Psychol.* **2006**, *3*, 77–101.

3. Alban, L.; Dahl, J.; Andreasen, M.; Petersen, J. V.; Sandberg, M. Possible impact of the "yellow card" antimicrobial scheme on meat inspection lesions in Danish finisher pigs. *Prev. Vet. Med.* **2013**, *108*, 334–341.

4. Cheng, A.C.; Turnidge, J.; Collignon, P.; Looke, D.; Barton, M.; Gottlieb, T. Control of Fluoroquinolone Resistance through Successful Regulation, Australia. *Emerg. Infect. Dis.* **2012**, *18*, 1453.

5. Clerc, O.; Prod'hom, G.; Senn, L.; Jaton, K.; Zanetti, G.; Calandra, T.; Greub, G. Matrix-assisted laser desorption ionization time-of-flight mass spectrometry and PCR-based rapid diagnosis of Staphylococcus aureus bacteraemia. *Clin. Microbiol. Infect.* **2014**, *20*, 355–360.

6. Altorf-Van Der Kuil[1], W.; Schoffelen[1], A.F.; De Greeff[1], S.C.; Ft Thijsen[2], S.; Alblas[1], J.; Notermans[3], D.W.; Lm Vlek, A.; Van Der Sande[1], M.A.; Leenstra[1], T. National laboratory-based surveillance system for antimicrobial resistance: a successful tool to support the control of antimicrobial resistance in the Netherlands Correspondence: Wieke Altorf-van der Kuil (Wieke.Altorf@rivm.nl). 1.

7. Deuster, S.; Roten, I.; Muehlebach, S. Implementation of treatment guidelines to support judicious use of antibiotic therapy. *J. Clin. Pharm. Ther.* **2010**, *35*, 71–78.

8. Fuertes, E.I.; Henry, B.; Marra, F.; Wong, H.; Patrick, D.M. Trends in Antibiotic Utilization in Vancouver Associated With a Community Education Program on Antibiotic Use. *Can. J. Public Health* **2010**, *101*, 304.

9. Fukuda, T.; Watanabe, H.; Ido, S.; Shiragami, M. Contribution of antimicrobial stewardship programs to reduction of antimicrobial therapy costs in community hospital with 429 Beds --before-after comparative two-year trial in Japan. *J. Pharm. Policy Pract.* **2014**, *7*, 1–7.

10. Fürst, J.; Čižman, M.; Mrak, J.; Kos, D.; Campbell, S.; Coenen, S.; Gustafsson, L.L.; Fürst, L.; Godman, B. The influence of a sustained multifaceted approach to improve antibiotic prescribing in Slovenia during the past decade: Findings and implications. *Expert Rev. Anti. Infect. Ther.* **2015**, *13*, 279–289.

11. Gaube, S.; Tsivrikos, D.; Dollinger, D.; Lermer, E. How a smiley protects health: A pilot intervention to improve hand hygiene in hospitals by activating injunctive norms through emoticons. **2018**.

12. Gottesman, B.S.; Carmeli, Y.; Shitrit, P.; Chowers, M. Impact of quinolone restriction on resistance patterns of Escherichia coli isolated from urine by culture in a community setting. *Clin. Infect. Dis.* **2009**, *49*, 869–875.

13. Hammerum, A.M.; Heuer, O.E.; Emborg, H.D.; Bagger-Skjøt, L.; Jensen, V.F.; Rogues, A.M.; Skov, R.L.; Agersø, Y.; Brandt, C.T.; Seyfarth, A.M.; et al. Danish Integrated Antimicrobial Resistance Monitoring and Research Program. *Emerg. Infect. Dis.* **2007**, *13*, 1633.

14. Hersberger, K.E.; Botomino, A.; Sarkar, R.; Tschudi, P.; Bucher, H.C.; Briel, M. Prescribed medications and pharmacy interventions for acute respiratory tract infections in Swiss primary care. *J. Clin. Pharm. Ther.* **2009**, *34*, 387–395.

15. Hiki, M.; Kawanishi, M.; Abo, H.; Kojima, A.; Koike, R.; Hamamoto, S.; Asai, T. Decreased Resistance to Broad-Spectrum Cephalosporin in Escherichia coli from Healthy Broilers at Farms in Japan After Voluntary Withdrawal of Ceftiofur. *https://home.liebertpub.com/fpd* **2015**, *12*, 639–643.

16. Huttner, B.; Harbarth, S. ' "Antibiotics Are Not Automatic Anymore"'-The French National Campaign To Cut Antibiotic Overuse.

17. Karp, B.E.; Tate, H.; Plumblee, J.R.; Dessai, U.; Whichard, J.M.; Thacker, E.L.; Hale, K.R.; Wilson, W.; Friedman, C.R.; Griffin, P.M.; et al. National antimicrobial resistance monitoring system: Two decades of advancing public health through integrated surveillance of antimicrobial resistance. *Foodborne Pathog. Dis.* **2017**, *14*, 545–557.

18. MacKinnon, M.C.; Pearl, D.L.; Carson, C.A.; Parmley, E.J.; McEwen, S.A. Comparison of annual and regional variation in multidrug resistance using various classification metrics for generic Escherichia coli isolated from chicken abattoir surveillance samples in Canada. *Prev. Vet. Med.* **2018**, *154*, 9–17.

19. Malla, S.; Dumre, S.P.; Shakya, G.; Kansakar, P.; Rai, B.; Hossain, A.; Nair, G.B.; Albert, M.J.; Sack, D.; Baker, S.; et al. The challenges and successes of implementing a sustainable antimicrobial resistance surveillance programme in Nepal. *BMC Public Health* **2014**, *14*, 1–7.

20. Meyer, E.; Lapatschek, M.; Bechtold, A.; Schwarzkopf, G.; Gastmeier, P.; Schwab, F. Impact of restriction of third generation cephalosporins on the burden of third generation cephalosporin resistant K. pneumoniae and E. coli in an ICU. *Intensive Care Med.* **2009**, *35*, 862–870.

21. Micallef, C.; Ashiru-Oredope, D.; Hansraj, S.; Denning, D.W.; Agrawal, S.G.; Manuel, R.J.; Schelenz, S.; Guy, R.; Muller-Pebody, B.; Patel, R.; et al. An investigation of antifungal stewardship programmes in England. *J. Med. Microbiol.* **2017**, *66*, 1581–1589.

22. Postma, M.; Vanderhaeghen, W.; Sarrazin, S.; Maes, D.; Dewulf, J. Reducing Antimicrobial Usage in Pig Production without Jeopardizing Production Parameters. *Zoonoses Public Health* **2017**, *64*, 63–74.

23. Nathwani, D.; Sneddon, J.; Malcolm, W.; Wiuff, C.; Patton, A.; Hurding, S.; Eastaway, A.; Seaton, R.A.; Watson, E.; Gillies, E.; et al. Scottish Antimicrobial Prescribing Group (SAPG): development and impact of the Scottish National Antimicrobial Stewardship Programme. *Int. J. Antimicrob. Agents* **2011**, *38*, 16–26.
